# Cuprous halides semiconductors as a new means for highly efficient light-emitting diodes

**DOI:** 10.1038/srep20718

**Published:** 2016-02-16

**Authors:** Doyeol Ahn, Seoung-Hwan Park

**Affiliations:** 1 Department of Electrical and Computer Engineering and Center for Quantum Information Processing, University of Seoul, Seoul 130-743, Republic of Korea; 2 Peta Lux Inc., 3F, TLi Building, 12 Yanghyeon-ro, 405 beon-gil, Jungwon-gu, Seongnam-si, Gyeonggi-do 462-100, Republic of Korea; 3 Electronics Department, Catholic University of Daegu, Hayang, Kyeongbuk 712-702, Republic of Korea

## Abstract

In group-III nitrides in use for white light-emitting diodes (LEDs), optical gain, measure of luminous efficiency, is very low owing to the built-in electrostatic fields, low exciton binding energy, and high-density misfit dislocations due to lattice-mismatched substrates. Cuprous halides I-VII semiconductors, on the other hand, have negligible built-in field, large exciton binding energies and close lattice matched to silicon substrates. Recent experimental studies have shown that the luminescence of I-VII CuCl grown on Si is three orders larger than that of GaN at room temperature. Here we report yet unexplored potential of cuprous halides systems by investigating the optical gain of CuCl/CuI quantum wells. It is found that the optical gain and the luminescence are much larger than that of group III-nitrides due to large exciton binding energy and vanishing electrostatic fields. We expect that these findings will open up the way toward highly efficient cuprous halides based LEDs compatible to Si technology.

Most of the research on white LEDs has been focused on the group-III nitride semiconductor based devices. However, it is well known^1^,^2^,^3^,^4^,^5^,^6^ that these devices employing nitride semiconductor quantum wells (QWs) show very low optical gain when compared with other III-V semiconductors such as GaAs due to the large built-in electrostatic fields on the order of MV/cm arising from the piezoelectric effects and the spontaneous polarizations. Also the large lattice mismatch between the nitride semiconductor and the substrates, typically, sapphire or SiC, leads to the generation of high density of misfit dislocations on the order of 10^10^ *cm*^−2^ which would also degrade the performances and the longevity of the device. In order to reduce the internal fields, an approach using the III-V wurtzite phase grown on non-polar or semi-polar substrates has been proposed^7^,^8^,^9^,^10^,^11^,^12^,^13^. However, layers grown on these non-polar or semi-polar substrates contain high density of non-radiative recombination centers, which have deleterious effects as well^13^. Another wide band-gap semiconductors such as II-VI ZnO has an energy band-gap of 3.3 eV at room temperature and an exciton binding energy of 63 meV^14^,^15^,^16^,^17^,^18^. For comparison, the exciton binding energy of GaN is 20 meV. The exciton binding energy is regarded as a measure of the interaction between electrons and holes and may be used to predict the strength of electron-hole recombination processes which are related to quantum efficiencies of the light emitting devices^19^. Therefore, wide band-gap II-VI ZnO quantum well (QW) structures have attracted much attention^14^,^15^,^16^. Unfortunately, it has proven difficult for ZnO semiconductor to achieve high p-type doping which is essential for the device implementation^17^.

Recently, I-VII *γ*-cuprous halides semiconductors^20^ such as CuCl, CuBr, and CuI have drawn attention^21^,^22^,^23^,^24^,^25^,^26^,^27^,^28^,^29^,^30^,^31^,^32^,^33^,^34^,^35^,^36^,^37^,^38^,^39^,^40^,^41^,^42^,^43^,^44^ because these are zincblende direct band-gap semiconductors (3.3 eV for CuCl, 2.91 eV for CuBr and 2.95 eV for CuI) and have large exciton binding energies (190 meV for CuCl, 108 meV for CuBr and 58 meV for CuI) with their lattice constants closely matched to that of Si as can be seen by the table 1. From this table, one can see that the lattice constant of Si, 0.543 nm, is very closely matched to that of CuCl, 0.542 nm. The cuprous halides atoms form tetrahedraly coordinated helides isomorphic with the diamond-crystal fcc lattice such as Si^45^. The zincblende structure of cuprous halides semiconductors consists of two interpenetrating fcc lattices displaced along a body diagonal. On one fcc lattice, the Cu atoms are located and on the other side the atoms are halogen type (Fig. 1). On top of that, these cuprous halides are transparent p-type in its natural states due to the presence of Cu vacancies resulting from excess halogen^42^,^43^,^44^. It is also shown^37^ that the incorporation of Zn by co-evaporation of CuCl and ZnCl_2_ yields n-type doping. The piezoelectric stress coefficient *e*_14_ for CuI is 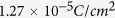 which is lower than that of GaAs, 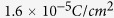 45,46. Since the piezoelectric effects of GaAs is much smaller than that of GaN or InGaN, we can ignore the piezoelectric field effects for CuI/CuCl QWs^47^. The spontaneous polarization arises from the intrinsic asymmetry of the bonding of wurtzite crystal structure^47^. Therefore, in the zincblende structure, the spontaneous polarization would be negligible.

Researches on the cuprous halides semiconductors have been focused on the following areas over the past decade: (1) spectroscopic and theoretical studies of band structures^26^,^27^,^28^,^29^,^30^,^31^,^32^,^33^, (2) photoluminescence studies of I-VII quantum dots embedded in NaCl crystals and glasses^22^,^24^,^25^,^33^, (3) surface studies of the growth mechanisms involved in the hetero epitaxy, and single crystal and poly crystal layer growth on Si and GaAs^23^,^35^,^36^,^37^,^38^,^39^,^40^. Especially, Nishida *et al.*^23^ demonstrated single crystal thin layera growth on GaAs and Si using ultra high vacuum (UHV) molecular beam epitaxy (MBE). As for a direct evidence of the exciton binding energy effects on the luminescence, it was observed that the luminescence of liquid phase epitaxy (LPE) grown polycrystalline CuCl on Si is considerably brighter (by 3 order of magnitude) than undoped single crystal GaN grown sapphire at room temperature^39^. An electroluminescence (EL) device employing polycrystalline *γ* -CuBr thin film active layer was also demonstrated^40^. However, there has been very little work on device physics studies of these I-VII semiconductors, considering their potential impacts on the high efficient light-emitting devices.

In this article, we report the theoretical study of an optical gain and the luminescence of I-VII CuI/CuCl quantum well structures on Si substrates in high efficiency light-emitting device for the first time. A multi-band effective mass approach^48^,^49^,^50^,^51^ and non-Markovian optical gain model including the excitonic effects are employed^52^,^53^. The Luttinger parameters of zincblende I-VII cuprous halides semiconductors, necessary for the band-structure calculation, are obtained from a semi-empirical five level 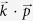 approach^41^,^48^,^49^. It is observed that the optical gain and the luminescence of cuprous halides CuI/CuCl and CuBr/CuCl QWs would be much higher than those of III-V nitride layers or II-VI ZnO/MgZnO QWs due to the inherent strong excitonic effects and negligible electrostatic fields within the active layers. Our predictions agree with recent experimental results^39^ qualitatively. Substantially high optical gain of I-VII cuprous halides QWs as compared with that of III-V nitride QWs or II-VI ZnO QWs and the cuprous halides semiconductor structure’s close lattice match to Si substrate are the clear manifestation of the possibility of highly efficient I-VII cuprous halides semiconductor based light-emitting devices for solid-state lighting and integrated optoelectronic components compatible to Si technology. This study is also expected to suggest further work on the device implementation of I-VII semiconductors.

## Results

We first obtain the Luttinger parameters of zincblende CuI and CuCl from a semi-empirical five level 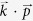 approach including d electron effects^41^,^48^,^49^. The band structure of a CuI/CuCl quantum well is calculated within the 6 × 6 multiband effective mass theory which also takes into account the biaxial strain, spontaneous polarization and the piezoelectric effects^13^,^41^. To calculate the optical gain, we used non-Markovian model based on time-convolutionless reduced-density operator formalism which includes the many-body effects such as the band-gap renormalization, enhancement of optical gain due to attractive electron-hole interaction called excitonic effects, and the plasma screening^52^,^53^. The mean field Coulomb effect is included in the interband reduced-density operator which gives the complete exciton effects with all bound states. Excitonic effects are particularly important for CuI and CuCl, which show the photoluminescence dominated by, *Z*_1,2_ and *Z*_3_ excitonic states in moderate carrier densities. In our model, the optical gain is given by^52^,^53^


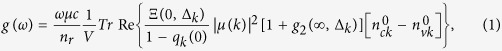


with


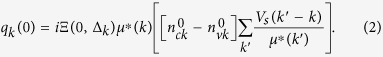


Here, *μ* is the permeability, *n*_*r*_ is the refractive index, *c* is the speed of light in free space, *V* is the volume, *Tr* denotes the trace, 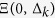 is the lineshape function that describes the spectral shape of the optical gain in driven semiconductor, *μ*(*k*) is the dipole moment, 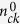 and 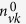 is the quasi-equilibrium distributions of electrons in the conduction band and valence band, respectively, *k* is the wave vector, *V*_*s*_(*k*) is screened Coulomb potential, *ω* is the angular frequency of the optical field, *g*_2_ is the optical phase detuning and 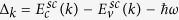, where 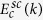 and 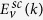 are renormalized energies of electrons in the conduction band and valence band, respectively.

In equation (1), the factor 1/(1 − *q*_*k*_(0)) describes the excitonic enhancement factor where the vertex function *q*_*k*_(0) is exact in the steady-state approximation and is equivalent to the one derived from the solution of the Bether-Salpeter equation obtained from the many-body Green’s function approach^52^. The excitonic effects are all contained in the vertex function *q*_*k*_(0).

### Band-structure

**Figure 2:**
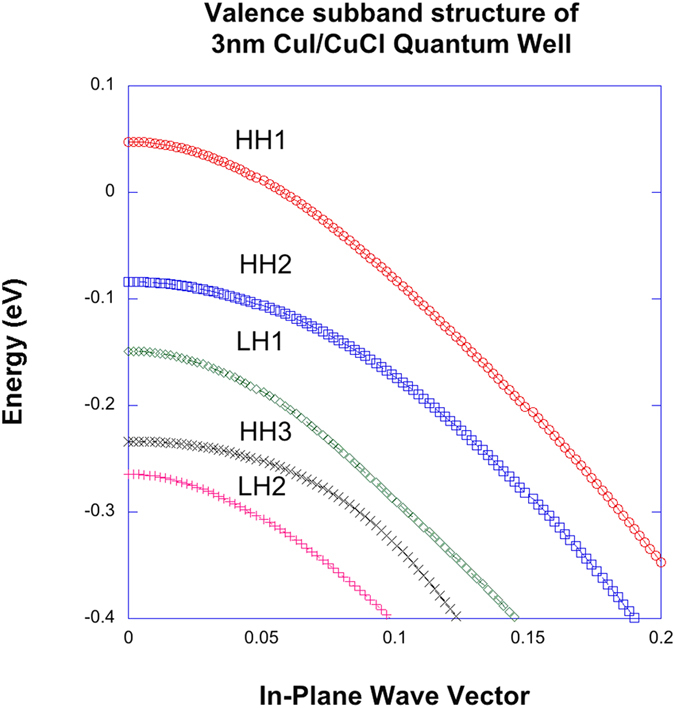
Valence-subband structure of a 30 Å CuI/CuCl quantum-well versus in-plane wave vector in unit of 2*π*/*a*_0_ where *a*_0_ is the lattice constant of CuI. We have used 6 × 6 Luttinger-Kohn model taking into account of the biaxial compressive strain due to the lattice mismatch between CuI/CuCl.

The results of our valence-subband calculation are shown in Fig. 2 for a 30 Å CuI/CuCl quantum-well versus in-plane wave vector in unit of 2*π*/*a*_0_ where *a*_0_ is the lattice constant of CuI. We have used 6 × 6 Luttinger-Kohn model taking into account of the biaxial compressive strain due to the lattice mismatch between CuI and CuCl. We assume that the band-gap discontinuity of CuI/CuCl quantum well is evenly distributed between the conduction band and the valence band. One must note that the spin-orbit (SO) band belong to Γ_7_ lies 40.4 meV above the Γ_8_ band in the case of CuCl but the SO band is below 640 meV from Γ_8_ band in the case of CuI at the Brillouin zone center^26^. As a result, the contribution of the SO band on the band mixing of heavy- and light-hole subbands would be negligible. In this figure, HH1 denotes the first state of the heavy hole (HH) subband and LH1 is the first state of light hole subbands.

### Optical gain and luminescence with excitonic enhancement

**Figure 3:**
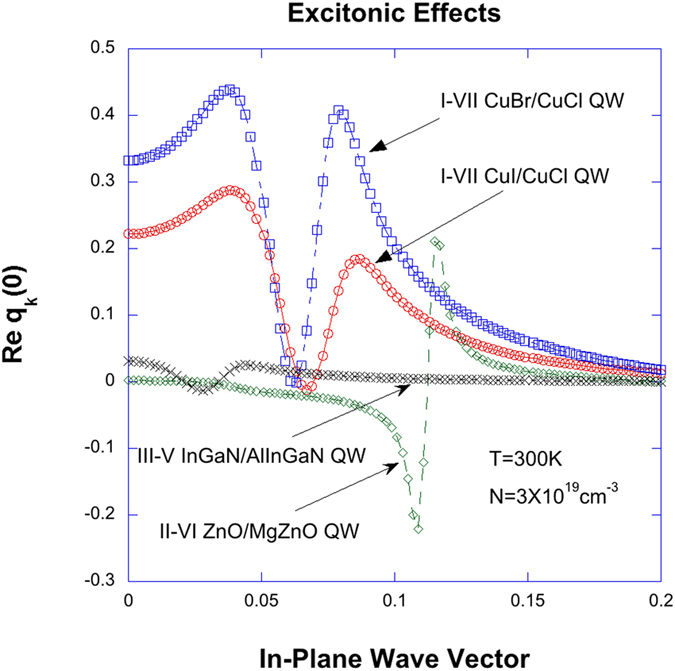
Re*q*_*k*_(0) between the ground states of conduction and valence bands for CuI/CuCl QW (red), CuBr/CuCl QW (blue), ZnO/Mg_0.3_Zn_0.7_O QW (green), and In_0.2_Ga_0.8_N/Al_0.2_In_0.005_Ga_0.7995_N QW (black). The carrier density of 3 × 10^19^ *cm*^−3^, intraband relaxation time of 10 fs and the correlation time of 25 fs are assumed in the calculation. In the cases of II-VI and III-V nitride QWs, the band structure of the hexagonal crystalline lattice is taken into account^13^.

From equation (1), it is evident that the integrand for the optical gain would be strongly affected by the excitonic enhancement factor 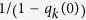. In Fig. 3, we show the Re*q*_*k*_(0) between the ground states of conduction and valence bands for CuI/CuCl QW (red), CuBr/CuCl QW^41^ (blue), ZnO/Mg_0.3_Zn_0.7_O QW (green), and In_0.2_Ga_0.8_N/Al_0.2_In_0.005_Ga_0.7995_N QW (black). The carrier density of 3 × 10^19^ *cm*^−3^, intraband relaxation time of 10 fs and the correlation time of 25 fs are assumed in the calculation^41^. In the cases of II-VI and III-V nitride QWs, the band structure of the hexagonal crystalline lattice is taken into account^13^. It is seen that the Re*q*_*k*_(0) for CuI/CuCl QW and CuBr/CuCl QW are much larger than that of InGaN/AlInGaN QW in magnitude when compared as functions of the in-plane wave vector. In all four cases, however, we have that Re*q*_*k*_(0) < 1. Since the Coulomb enhancement factor is inversely proportional to 1 − Re*q*_*k*_(0), the excitonic effects on the optical gain would be appreciable for I-VII QWs as can be seen in this figure. From this result, it is predicted that the enhancement of gain would be most pronounced in the case of I-VII QW then followed by II-VI ZnO/MgZnO QW. The strong excitonic effects manifested by Re*q*_*k*_(0) in Fig. 2 agree at least qualitatively with the experimental results^32^,^33^,^34^.

**Figure 4:**
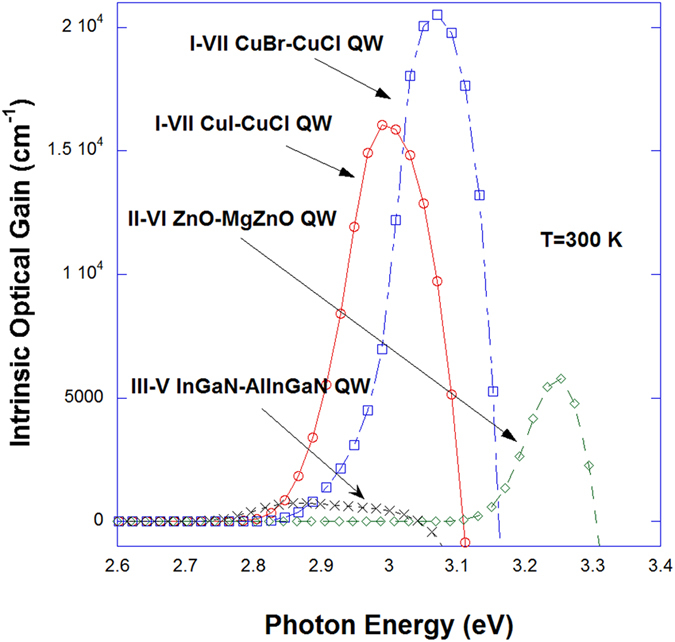
on-Markovian optical gain spectra with Coulomb or excitonic enhancement are plotted for CuI/CuCl QW (red), CuBr/CuCl QW (blue), ZnO/Mg_0.3_Zn_0.7_O QW (green), and In_0.2_Ga_0.8_N/Al_0.2_In_0.005_Ga_0.7995_N QW (black) versus photon energy for carrier density of 5 × 10^19^ *cm*^−3^. Band-gap renormalization is taken into account in all cases. In the cases of II-VI and III-V nitride QWs, the band structure of the hexagonal crystalline lattice is taken into account^13^.

**Figure 5:**
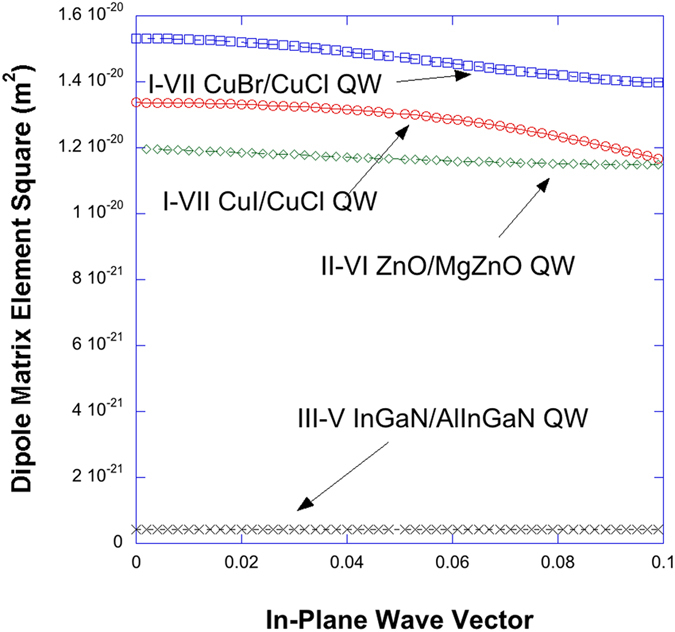
Dipole matrix element squares are plotted for CuI/CuCl QW (red), CuBr/CuCl QW (blue), ZnO/Mg_0.3_Zn_0.7_O QW (green), and In_0.2_Ga_0.8_N/Al_0.2_In_0.005_Ga_0.7995_N QW (black) versus photon energy. In the cases of II-VI and III-V nitride QWs, the band structure of the hexagonal crystalline lattice is taken into account (ref. 13).

In Fig. 4, non-Markovian optical gain spectra with Coulomb or excitonic enhancement are plotted for CuI/CuCl QW (red), CuBr/CuCl QW (blue), ZnO/Mg_0.3_Zn_0.7_O QW (green), and In_0.2_Ga_0.8_N/Al_0.2_In_0.005_Ga_0.7995_N QW (black) versus photon energy for carrier density of 5 × 10^19^ *cm*^−3^. Band-gap renormalization is taken into account in all cases. From Figs 3 and 4, we expect that the many-body effects, especially, the Coulomb effects are becoming more important in the case of cuprous halides I-VII QWs whose peak gain is an order of larger than that of InGaN-AlInGaN QW. The optical gain of II-VI ZnO/MgZnO QW is still larger than that of InGaN-AlInGaN QW but ZnO cannot be grown on Si because of too much lattice mismatch unlike the case of I-VII QWs and p-type doping is difficult. I-VII CuI and CuBr are known to have similar effective masses as GaN at the zone center^35^, so the main reason of much larger gain of the cuprous halides semiconductor is the large diploe matrix elements which is almost an order of magnitude larger than that of nitride semiconductors as can be seen in Fig. 5 and the excitonic effects shown in Fig. 3.

The dipole moment 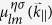 is defined by^52^


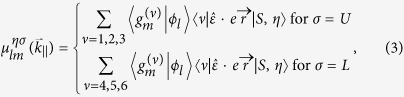


where 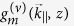 is the hole envelope function; 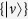 denotes the transformed Bloch basis at the zone center; m is the quantum well subband index; 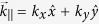, 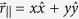 and *σ* = *U*(or *L*) refers to the upper (or lower) blocks; respectively; *ϕ*_*l*_(*z*) is the electron envelope function for the *l*th conduction subband with a spin state *η* ; and 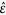 is the unit vector in the direction of the photon polarization.

**Figure 6:**
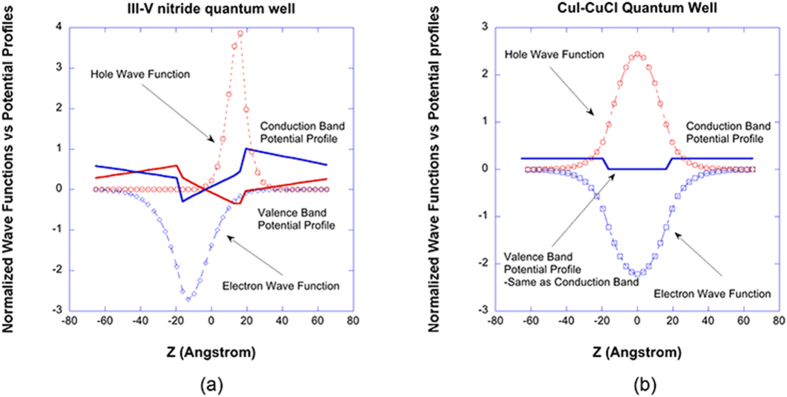
Normalized ground state electron wave functions (blue) and the hole wave functions (red) at the zone center are plotted together with the conduction and valence band QW potential profiles for (**a**) In_0.2_Ga_0.8_N/Al_0.2_In_0.005_Ga_0.7995_N QW and (**b**) CuI/CuCl QW.

From equation (3), it is straightforward to see that the dipole moment is proportional to the overlap integral of electron and hole wave functions. In Fig. 6, normalized ground state electron wave functions (blue) and the hole wave functions (red) at the zone center are plotted together with the conduction and valence band QW potential profiles^54^ for (a) In_0.2_Ga_0.8_N/Al_0.2_In_0.005_Ga_0.7995_N QW and (b) CuI/CuCl QW. In the case of III-V nitride QW, the built-in electrostatic fields cause the electron wave function and the hole wave function shifted to the opposite side of the QW, thus reducing the overlap integral in equation (3) significantly. The vertex function *q*_*k*_(0) for the excitonic can be approximated as^52^


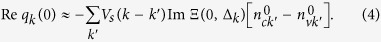


where the screened Coulomb potential *V*_*s*_(*k*) is given by^52^


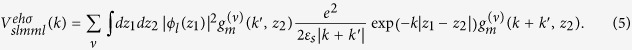


From equation (5), one can see that the excitonic enhancement is also strongly dependent on the overlap between the electron and hole wave functions.

**Figure 7:**
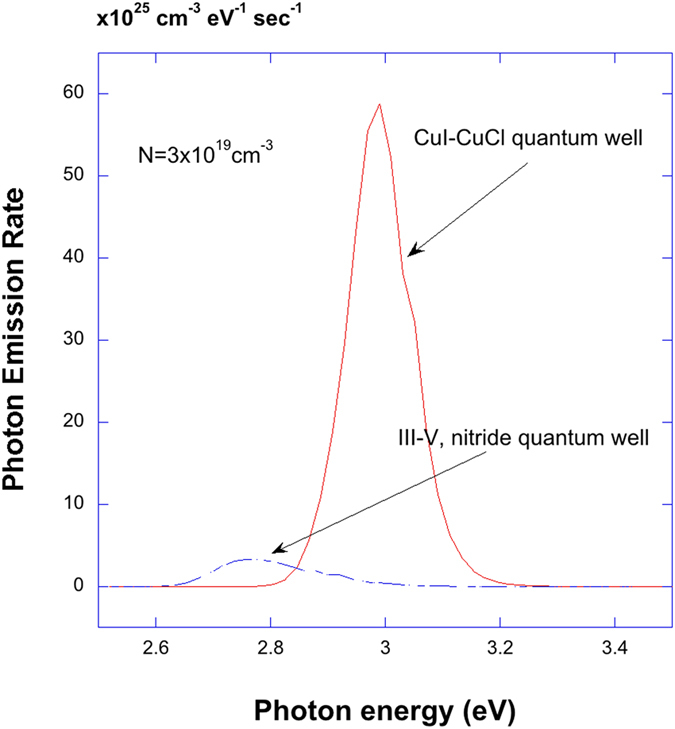
Luminescence spectra which gives the photon emission rate are plotted for CuI/CuCl QW (red) and In_0.2_Ga_0.8_N/Al_0.2_In_0.025_Ga_0.7975_N QW (blue) versus photon energy for the carrier density of 3 × 10^19^ *cm*^−3^. The luminescence is described modified van Roosbroeck-Shockley model^52^,^53^,^55^.

**Figure 8:**
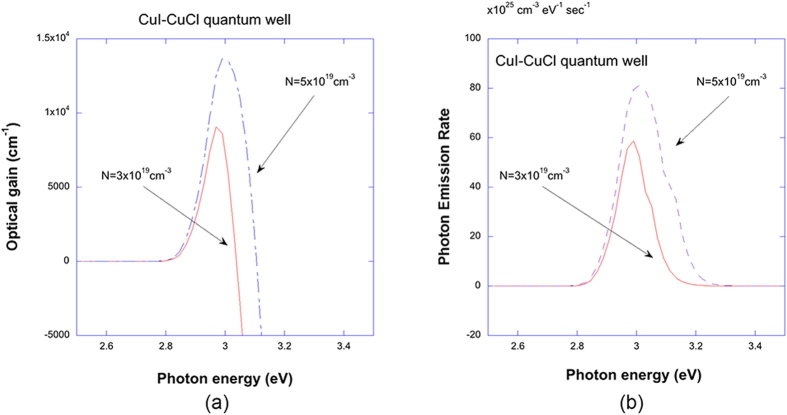
(**a**) Non-Markovian optical gain spectra with Coulomb or excitonic enhancement are plotted for CuI/CuCl QW versus photon energy for carrier densities of 3 × 10^19^ *cm*^−3^ (red) and 5 × 10^19^ *cm*^−3^ (blue). (**b**) Non-Markovian lumiescence spectra with Coulomb or excitonic enhancement are plotted for CuI/CuCl QW versus photon energy for carrier densities of 3 × 10^19^ *cm*^−3^ (red) and 5 × 10^19^ *cm*^−3^ (blue).

From the LED device point of view, one needs to convert the optical gain into the luminescence^55^,^56^,^57^,^58^ which describes the radiative recombination rate under equilibrium and non-equilibrium conditions. The luminesce is described by the spontaneous emission rate *R*_*sp*_(*ω*), the number of emitted photons per second per unit volume per unit energy interval, is related to the optical gain *g*(*ω*) by^56^,^57^,^58^


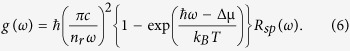


where 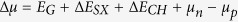 ; *μ*_*n*_ and *μ*_*p*_ are renormalized chemical potentials for the electron and the hole, respectively, such that *g*(Δ*μ*) = 0; *k*_*B*_ is the Boltzmann constant; and *T* is the temperature. We note that at 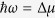, the definition of *R*_*sp*_(*ω*) breaks down, so we interpolated *R*_*sp*_(*ω*) at 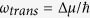 from the values of *R*_*sp*_(*ω*) at 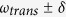. The importance of using the non-Markovian lineshape functions would be pronounced in the above relation between the spontaneous emission rate and the optical gain. One of the remarkable feature of this relation is that there is a transparency point in the gain spectra which coincide with the chemical potential separation that suggests the carriers and the photons are in equilibrium or in quasi-equilibrium^57^. The optical gain spectra calculated with the Lorentzian line shape function have two errors: unnatural absorption region below the renormalized bandgap energy and mismatch of the transparency point of the gain with the chemical potential separation. It was shown in the previous work^57^, that these two anomalies associated with the Lorentzian lineshape are removed in the non-Markovian model with many-body effects. In Fig. 7, the luminescence spectra calculated by equation (5) are plotted for CuI/CuCl QW (red) and In_0.2_Ga_0.8_N/Al_0.2_In_0.025_Ga_0.7975_N QW (blue) versus photon energy for the carrier density of 3 × 10^19^ *cm*^−3^. From Fig. 7, it is expected that an order of magnitude increase for cuprous halides as compared with group III-nitride quantum wells. The efficiency of the luminescence for LEDs would depend on the competition of radiative and non-radiative processes and detailed analysis of quantum efficiency of cuprous halides based LEDs would need further study. In Fig. 8, both optical gain and luminescen spectra for CuI-CuCl QW are calculated for different carrier densities.

In this work, we focused on cuprous haldes especially, CuI-CuCl system. There are also transition metal halides such as ZnCl_2_. In cuprous halides, the loosely bound s electrons of the Cu atom is mostly transferred to the more electronegative halogen^59^. This leaves the Cu ion with completely filled outer d shell and the halogen ion with the rare-earth configuration. Unlike the I-VII alkali halides in which the d shell are core-like, the spatial extent of the d level is large and their energies are close to those of the p levels of the halogen^59^. On the other hand, most transition metal has partially filled d shells except Zn which has complexly filled d shell with the electronic configuration of d^10^s^2^. If the d shell of Zn ion is not core-like and the comparable to p level energies of the halogen atom, then its behaviour may be similar to that of cuprous halides. Otherwise, electronic as well as optical properties would be different.

It would require further work to compare the cuprous halides and transition metal halides.

## Discussions

Built-in electrostatic fields in the active layer of the group-III nitrides LEDs have deleterious effects on the luminous efficiencies^1^,^2^,^3^,^4^,^5^,^6^. It is also found that the use of lattice-mismatched substrates cause the generation of high-density misfit-dislocations that affect the longevity of the device. Several attempts including the use of non-polar^7^,^8^,^9^,^10^,^11^,^12^,^13^ substrates are being tried with varying degree of success. In the present article, we have reported yet unexplored potential of cuprous halides semiconductors for highly efficient LEDs. Our predictions agree with recent experimental results^39^ at least qualitatively. Expected high performance of cuprous halides system is due to large exciton binding energy, vanishing electrostatic field in the active layer and close lattice match with the substrate, silicon. Considering that the application of cuprous halides semiconductors to optoelectronic devices is still in very early stage of research and development, we expect that our results reported in this article may have significant impacts on the future optoelectronic device technologies.

## Additional Information

**How to cite this article**: Ahn, D. and Park, S.-H. Cuprous halides semiconductors as a new means for highly efficient light-emitting diodes. *Sci. Rep.* **6**, 20718; doi: 10.1038/srep20718 (2016).

## Figures and Tables

**Figure 1:**
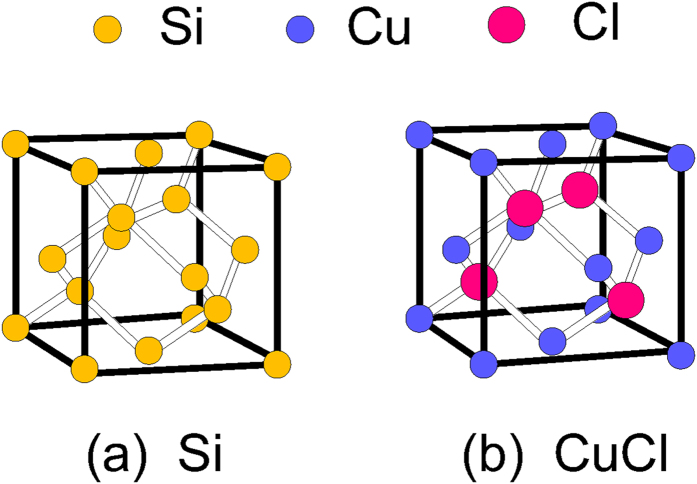
(**a**) The diamond-crystal fcc lattice characterized by four covalent bonded Si atoms. (**b**) The zincblende fcc lattice of cuprous halides crystals such as CuCl, CuBr and CuI. The zincblende structure consists of two interpenetrating fcc lattices displaced along a body diagonal. On one fcc lattice, the atoms are Cu and on the other side they are halogen atoms.

**Table 1:** Material parameters of cuprous halides semiconductors (ref. 45).

	CuCl	CuBr	CuI
*E*_*g*_(*eV*)	3.399	2.91	2.95
*m*_*c*_(Γ_6*C*_)/*m*_0_	0.5	0.21	0.3
*m*_*p*_(Γ_7*V*_)/*m*_0_	2.0	1.6	2.43
Lattice constant (nm)	0.54202	0.56897	0.60521
*C*_11_(10^11^ *dyn cm*^−2^)	0.47	0.458	0.451
*C*_12_(10^11^ *dyn cm*^−2^)	0.362	0.354	0.307
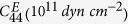	0.145	0.139	0.182
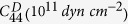	0.162	–	0.185
*b*(*eV*)	−0.7	−0.4	–
*ε*(∞)	7.9	4.062	4.58
Δ_*so*_(*meV*)	−40.4	150	640
Exciton binding energy (*meV*)	190	108	58
